# Phase Evolution and Mechanical Performance of Zirconia Ceramics Synthesized Under High Temperature and High Pressure

**DOI:** 10.3390/nano15161235

**Published:** 2025-08-13

**Authors:** Jin You, Wenjie Guo, Yangyang Li, Yilong Pan, Tian Cui

**Affiliations:** Institute of High Pressure Physics, School of Physical Scientific and Technology, Ningbo University, Ningbo 315211, China; youjin0120@163.com (J.Y.);

**Keywords:** high temperature and high pressure, zirconia, Vickers hardness, fracture toughness, phase transition

## Abstract

Achieving simultaneous enhancement of Vickers hardness and fracture toughness remains a critical challenge in designing oxide ceramic materials due to their partially antagonistic nature. In this study, we address this trade-off by tailoring the microstructure of zirconia (ZrO_2_) ceramics through Y_2_O_3_ doping and high-pressure high-temperature (HPHT) sintering. Nanostructured composites were synthesized using 50 nm monoclinic ZrO_2_ and varying Y_2_O_3_ contents (3, 5, and 7 mol%) under 5 GPa at temperatures ranging from 400 to 2000 °C. Among them, 3 mol% Y_2_O_3_-doped zirconia (3Y-PSZ) sintered at 1200 °C achieved a well-balanced mechanical performance, with a Vickers hardness of 11.6 GPa and a fracture toughness of 9.26 MPa·m^1/2^. These results demonstrate that it is feasible to retain a high hardness while significantly enhancing toughness by controlling phase composition and grain refinement under HPHT conditions. This work offers valuable insights into microstructural optimization strategies for zirconia-based ceramics aiming to overcome the conventional hardness–toughness trade-off.

## 1. Introduction

ZrO_2_ has garnered extensive attention across various fields, including bioceramics, cutting tools, refractories, and solid oxide fuel cells, owing to its outstanding mechanical properties and chemical stability [1,2,3,4]. At ambient conditions, ZrO_2_ adopts the monoclinic (m) phase, while the tetragonal (t) and cubic (c) phases are stable only at elevated temperatures [5,6,7]. The m-phase exhibits inferior toughness and strength, whereas the t- and c-phase display enhanced mechanical performance. However, the transformation from the t- to m-phase during cooling leads to a volumetric expansion (~3–5%), causing microcracks and catastrophic material failure [8]. To address this challenge, stabilizing oxides such as Y_2_O_3_, CeO_2_, MgO, and CaO are introduced to promote the metastable retention of t- and c-phase, facilitating transformation toughening [9,10,11,12,13,14,15,16]. Among these, yttria-stabilized zirconia (Y-PSZ) stands out due to its favorable combination of hardness, fracture toughness, and biocompatibility, which has led to its widespread use in dental restorations, orthopedic implants, and advanced ceramic cutting tools [17]. For instance, Alhotan et al. utilized a lithography-based ceramic manufacturing process to fabricate 3D-printed 3Y-TZP materials, achieving a fracture toughness of 6.07 MPa∙m^1/2^ and a hardness of 14.2 GPa after multistep thermal treatments [18]. Similarly, Tovar-Vargas et al. reported that a composite of 10 mol% Ce-TZP and 1 mol% CaO sintered at 1450 °C under atmospheric pressure achieved a fracture toughness of 6.5 MPa∙m^1/2^ and hardness up to 9.9 GPa [19].

Despite the significant progress in stabilizing ZrO_2_, achieving a favorable balance between high hardness and enhanced fracture toughness remains a critical scientific challenge. Various strategies have been proposed to overcome this trade-off, including the optimization of dopant content, grain size refinement, the incorporation of second-phase reinforcements (e.g., Al_2_O_3_, SiC), and the adoption of advanced sintering techniques. HPHT sintering has emerged as a particularly effective approach for enhancing mechanical performance. Elevated pressure can suppress long-range atomic diffusion and inhibit grain coarsening, leading to an increase in grain boundary density and a reduction in dislocation mobility. This in turn improves yield strength, tensile strength, and fracture toughness [20,21]. Moreover, the application of high pressure facilitates densification, thereby further contributing to the enhancement of mechanical properties [22,23].

Previous studies have shown that high pressure significantly accelerates the phase transition kinetics of ZrO_2_, thereby enhancing its structural evolution [24,25]. For example, Vahldiek et al. synthesized the t-phase at 1.5–2 GPa and 1200–1700 °C [26], while Kulcinski demonstrated that the t-phase could be formed directly from the m-phase at room temperature under pressures exceeding 3.7 GPa, although the transformation was entirely reversible upon pressure release [27]. Despite advances in understanding ZrO_2_’s phase transitions under pressure, limited attention has been paid to simultaneously optimizing both hardness and fracture toughness.

To bridge this gap, this study systematically investigates the effects of varying Y_2_O_3_ content (3, 5, and 7 mol%) and sintering temperatures (400–2000 °C) under 5 GPa on the microstructure and mechanical properties of ZrO_2_ ceramics. A ZrO_2_ bulk ceramic exhibiting maximized fracture toughness without compromising hardness was successfully achieved by sintering at 1200 °C with 3 mol% Y_2_O_3_ doping. This work provides new insights into the design of high-performance ZrO_2_-based ceramics under high-pressure conditions and offers a theoretical foundation for the development and application of advanced ceramic materials.

## 2. Materials and Methods

**Figure 1:**
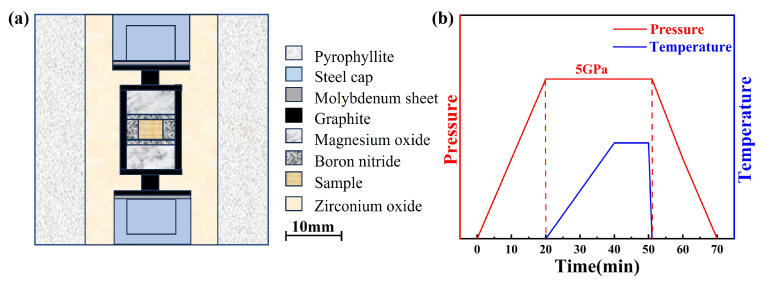
(**a**) Schematic diagram of the experimental assembly; (**b**) schematic of the experimental temperature and pressure program.

The ZrO_2_ ceramics were consolidated via HPHT sintering using a Chinese domestic large-volume cubic press (GY560, SPD-6 × 2700 kN, Guilin Guiye Machinery Co., Ltd., Guilin, China) under a constant pressure of 5 GPa and within a temperature range of 400–2000 °C. m-ZrO_2_ nanopowders with an average particle size of ~50 nm (purity ≥ 99.99%, Beijing Innochem Technology Co., Ltd., Beijing, China) and Y_2_O_3_ powders (purity ≥ 99.99%, Beijing Innochem Technology Co., Ltd.) were used as starting materials. Y_2_O_3_ was added to the ZrO_2_ powder in stoichiometric amounts to achieve doping levels of 3, 5, and 7 mol%. The well-mixed powder was uniaxially pre-pressed under 10 MPa into cylindrical compacts with a diameter of 6 mm, which were then loaded into boron nitride (BN) ceramic tubes for sintering. The heating assembly consisted of a BN tube serving as a high-pressure liner, a graphite tube as the heating element, and pyrophyllite as the pressure-transmitting medium (all purchased from Zibo Jingyi Ceramics Co., Ltd., China, Zibo, China). Prior to assembly, all components were baked at 100 °C for 12 h to remove residual moisture. A schematic diagram of the assembly setup is shown in Figure 1a, while the experimental pressure–temperature profile is illustrated in Figure 1b. The pressure was ramped to 5 GPa within 20 min, and the temperature was raised to the target value over another 20 min, followed by a 10-min dwell time at the set temperature. The experimental synthetic pressure was based on the high-pressure phase transformation point of Bi, Ba, and Tl, and the chamber temperature was calibrated by a high-pressure in situ resistance measurement and monitored using Pt-30%Rh/Pt-6%Rh (0–1800 °C) and W-Re3/25 (1800–2300 °C) thermocouples.

Phase composition was characterized by X-ray diffraction (XRD, D8 ADVANCE, Bruker, Ettlingen, Germany, Cu-Kα radiation, and λ = 0.15418 nm). Raman spectra were obtained using the Monovista CRS+ (Monovista CRS+, Spectros)equipped with a 532 nm laser. Microstructure and grain morphology were examined using the Scanning Electron Microscope (FE-SEM, SU-70, Hitachi High-Technologies, Tokyo, Japan). Grain size distributions were determined by analyzing 100 g from SEM images. Vickers hardness (HV) was measured under a load of 19.6 N with a dwell time of 10 s using an HMAS-D tester (Shanghai Microcre Light-Mach Tech Co., Ltd., Shanghai, China), while fracture toughness was calculated based on crack lengths obtained under higher loads (>19.6 N) using a KB5 tester (KB Prüftechnik, Hochdorf-Assenheim, Germany). Young’s modulus was determined using a nanoindenter (G200, KLA Corporation, Chandler, AZ, USA) under a maximum load of 1000 mN. The HV [14] and fracture toughness (K_IC_) [28] were calculated using the corresponding standard equations:

(1) $$HV=\frac{1885.4P}{d^{2}}$$

(2) $$K_{IC}=0.004985{\left(\frac{E}{HV}\right)}^{1/2}\frac{P}{c^{3/2}}$$

where *P* is the applied load (Kg), *d* is the average diagonal length (mm) of the indentation, *c* is the average length (mm) of the radial cracks, and *E* (230 GPa) is the elastic modulus of the ZrO_2_-based ceramic composites.

## 3. Results and Discussion

### 3.1. Structure and Surface Morphology

**Figure 2:**
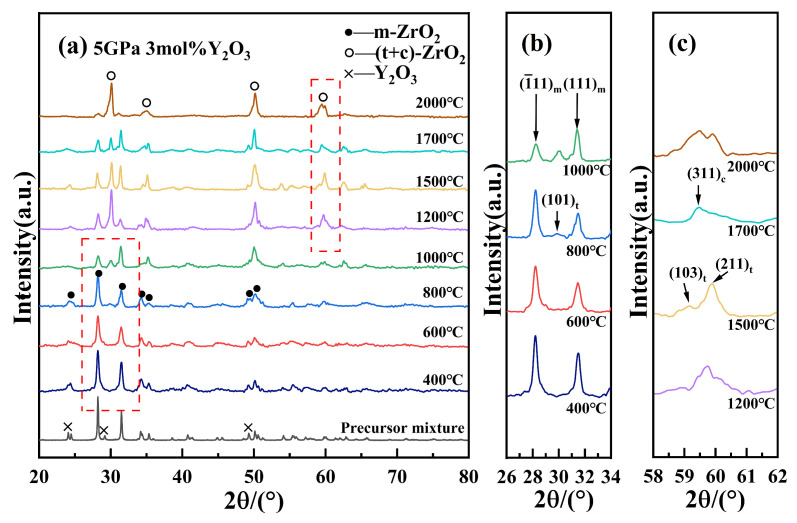
(**a**) XRD patterns of 3Y-PSZ at various temperatures at 5 GPa; (**b**) partial enlargement of the (101)_t_ diffraction peak; and (**c**) the high-angle region of the XRD patterns (around 2θ ≈ 60°).

Figure 2 presents the XRD patterns of 3Y-PSZ synthesized under 5 GPa at various temperatures. As shown in Figure 2a, with the increasing sintering temperature from 400 °C to 2000 °C, the intensities of the t- and c-phase peaks gradually increased, while the m-phase peaks weakened, suggesting a progressive phase transformation from the m-phase to the t- and c-phase. When 3Y-PSZ is sintered above 1000 °C, Y^3+^ ions are likely to fully dissolve into the ZrO_2_ lattice, stabilizing either the t- or c-phase. As a result, the absence of distinct Y_2_O_3_ diffraction peaks in the XRD pattern is plausible. A localized enlargement of the 26–34° region, displayed in Figure 2b, shows that the weak t-phase peaks [29] appeared at 2θ ≈ 30.3° after 800 °C. Between 800 °C and 1500 °C, the t-phase fraction steadily increased from 31% to 62%, as calculated by the ratio of integrated intensities of selected reflections corresponding to the m-, t-, and c-phases, respectively. After 1700 °C, a new diffraction peak corresponding to the (311) plane of the c-phase emerged (Figure 2c), indicating the coexistence of m-, t-, and c-phase. At 2000 °C, the combined fraction of t- and c-phase increased significantly, with the c-phase becoming more dominant and the total high-symmetry phase content (t + c) reaching approximately 70%. Table 1 summarizes the sintering temperature, pressure, holding time, phase composition, and phase fractions for the 3Y-PSZ samples.

**Figure 3:**
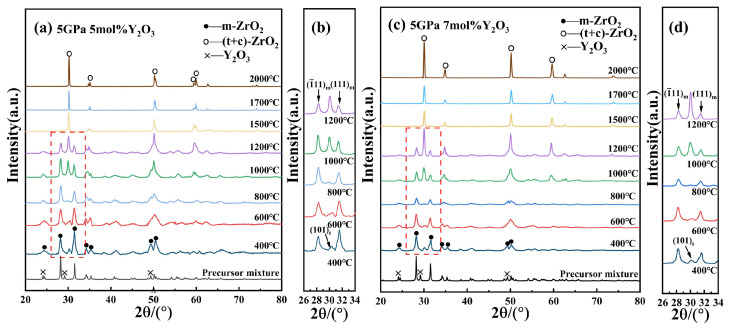
(**a**,**b**) XRD patterns of 5Y-PSZ at different temperatures under 5 GPa and enlarged views of m- and t-phase peaks; (**c**,**d**) XRD patterns and enlarged views of 7Y-PSZ at different temperatures under 5 GPa and enlarged views of m- and t-phase peaks.

As shown in Figure 3, the XRD results for 5 mol% and 7 mol% Y-PSZ samples (5Y-PSZ, 7Y-PSZ) reveal similar phase evolution trends. Notably, the t-phase first appeared at 400 °C in both samples, indicating that higher Y_2_O_3_ content lowers the onset temperature for phase stabilization. By 1500 °C, m-phase peaks disappeared entirely, indicating a complete transformation into t- and c-phases. This further verifies that the increasing Y_2_O_3_ concentration effectively reduces the temperature required for the m- to c-phase transition. The progressive enhancement of t- and c-phase peaks, along with the gradual fading of m-phase peaks with the increasing temperature, further confirms the high-pressure-assisted phase transformation toward more stable high-symmetry phases. This phenomenon is attributed to the substitution of Zr^4+^ by larger Y^3+^ ions, which generates oxygen vacancies to maintain charge neutrality. The vacancies disrupt the long-range order of the oxygen sublattice and promote structural disorder, thereby reducing the free energy barrier for the formation of high-symmetry phases. In addition, lattice expansion caused by Y^3+^ incorporation helps to alleviate internal strain, making the c-phase thermodynamically favorable at lower temperatures under HPHT conditions.

**Figure 4:**
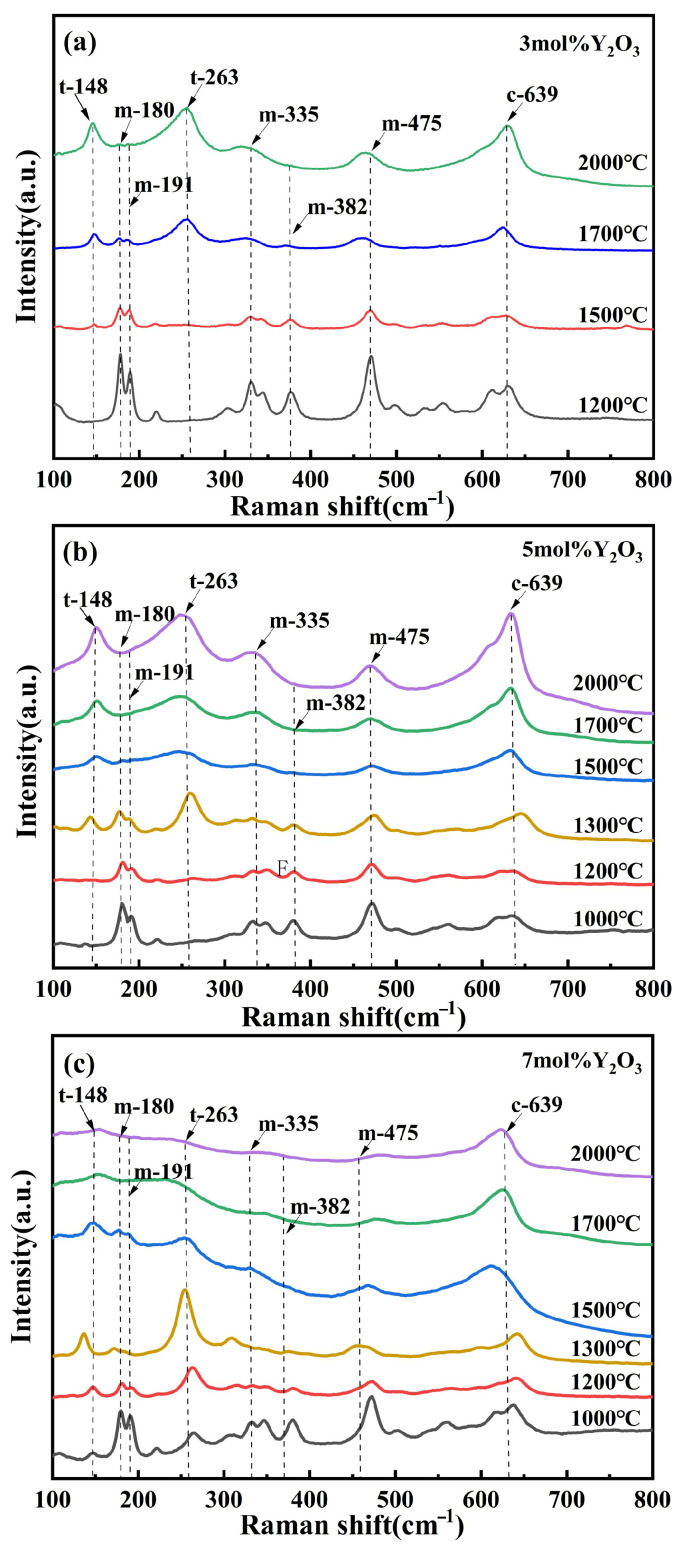
(**a**–**c**) Present the Raman spectra of (3, 5, and 7)Y-PSZ, respectively, synthesized under a pressure of 5 GPa and sintering temperatures ranging from 1000 to 2000 °C.

Due to the overlap of the (101)_t_ and (111)_c_ diffraction peaks and the highly similar lattice parameters of the t- and c-phase, conventional XRD characterization cannot effectively differentiate between these two structures [30]. Therefore, Raman spectroscopy was employed to further analyze the phase composition of the ZrO_2_ samples. The t- to c-phase transformation is primarily influenced by the relative displacement of one crystallographic axis compared to the other two, along with adjustments of the oxygen ion positions within the fluorite structure [31]. Notably, the Raman spectrum of the c-phase exhibits a broad band between 530 and 670 cm^−1^, which is distinct from the sharp peaks characteristic of the m- and t-phase [32]. Figure 4 shows the Raman spectra of (3, 5, and 7)Y-PSZ samples, respectively, synthesized at temperatures ranging from 1000 °C to 2000 °C under 5 GPa. Sharp Raman peaks at m-180 cm^−1^, m-191 cm^−1^, and m-475 cm^−1^, along with strong peaks at ~335 cm^−1^, ~348 cm^−1^, and ~382 cm^−1^ (as shown by the labeled peaks in the figures) are characteristic of the m-phase, while the peaks at t-148 cm^−1^ and t-263 cm^−1^ are unique to the t-phase [33,34]. In Figure 4a, the intensity of the m-phase peaks (m-180 cm^−1^, m-191 cm^−1^, and m-475 cm^−1^) decreases with the increasing sintering temperature from 1200 °C to 1500 °C, while the t-phase peaks at (148 cm^−1^ and 263 cm^−1^) become pronounced. This trend indicates a progressive phase transformation from m- to t-phase. The c-phase is characterized by a broad Raman band in the 530–670 cm^−1^ region. In Figure 4a, this band becomes noticeably broader at 1700 °C compared to 1500 °C, which is consistent with the emergence of a c-phase (311)_c_ diffraction peak in the corresponding XRD patterns. At 2000 °C, the intensities of the m-phase peaks further decrease, while the combined fraction of t- and c-phase increases from 47% to 70%, resulting in a three-phase composition. Similarly, Raman broadening is observed at 1500 °C for 5Y-PSZ (Figure 4b) and 1200 °C for 7Y-PSZ (Figure 4c), confirming the formation of the c-phase at lower temperatures with higher Y_2_O_3_ content. These results suggest that the formation of the c-phase is significantly promoted under 5 GPa, and the critical temperature for its appearance decreases with increasing Y_2_O_3_ content; approximately 1700 °C for 3Y-PSZ, 1500 °C for 5Y-PSZ, and 1200 °C for 7Y-PSZ.

**Figure 5:**
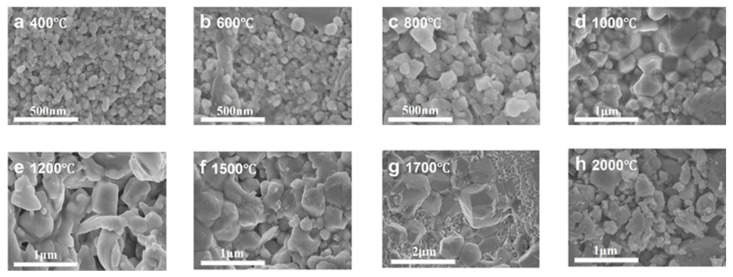
SEM images of 3Y-PSZ synthesized under 5 GPa at temperatures ranging from 400 to 2000 °C.

**Figure 6:**
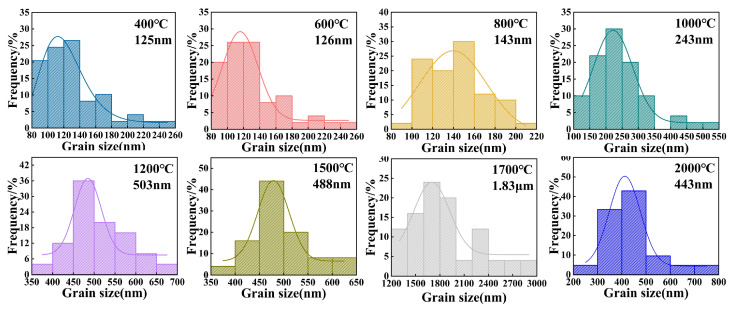
Grain size distributions of 3Y-PSZ synthesized under 5 GPa at temperatures ranging from 400 to 2000 °C.

Figure 5 and Figure 6 present the microstructural evolution and grain size distributions of 3Y-PSZ synthesized under 5 GPa. SEM images (Figure 5) reveal dense, crack-free microstructures with a homogeneous dispersion of the m- and t-phase. Grain size analysis (Figure 6) shows minimal growth at 400–600 °C (from 125 nm to 126 nm), consistent with the absence of t-phase XRD signals (Figure 2), confirming the phase stability below 800 °C. At 800 °C, the emergence of the t-phase is accompanied by a moderate grain coarsening to 143 nm. Further heating to 1000 °C leads to distinct t-phase stabilization and accelerated grain growth (243 nm) driven by enhanced atomic diffusion and grain boundary migration driven by interfacial energy minimization. Progressive heating to 1200–2000 °C yields non-monotonic growth: 503 nm at 1200 °C, 488 nm at 1500 °C, and 1.83 μm at 1700 °C, followed by an anomalous refinement to 443 nm at 2000 °C. This reversal suggests a competitive interplay between thermal-induced coarsening and high-pressure-induced lattice strain, highlighting the complex balance between thermodynamic and mechanical factors in extreme-condition sintering.

### 3.2. Mechanical Properties

**Figure 7:**
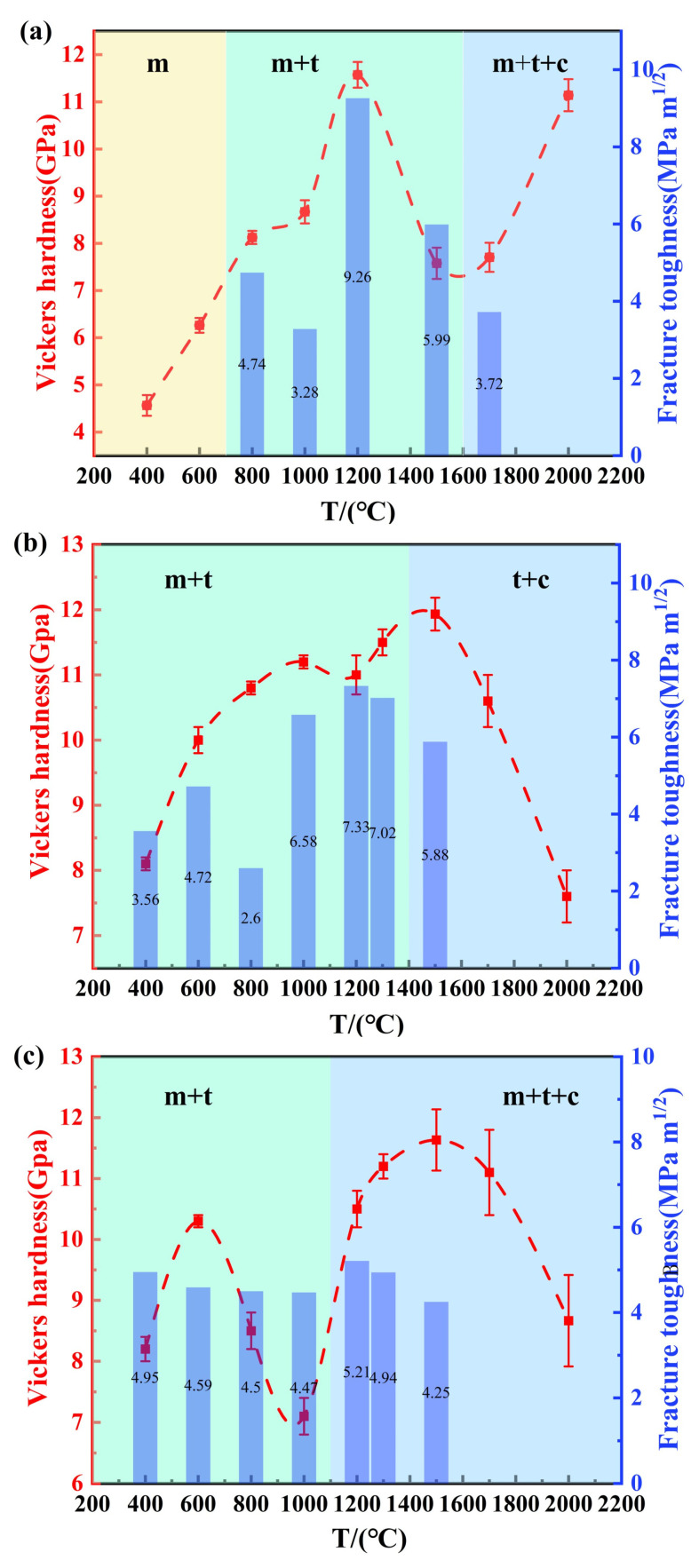
(**a**–**c**) Average Vickers hardness (left-axis) and fracture toughness (right-axis) of (3, 5, and 7)Y-PSZ synthesized at different temperatures under 5 GPa.

Figure 7 presents the Vickers hardness (under a load of 19.6N) and fracture toughness of (3, 5, and 7)Y-PSZ, respectively, synthesized at different temperatures under 5 GPa. For 3Y-PSZ (Figure 7a), the samples sintered at 400 °C and 600 °C remained in the m-phase with slightly coarsened grains, showing low-average hardness values of 4.6 GPa and 6.6 GPa. With the increasing temperature, both hardness values and fracture toughness improved, reaching maximum values of 11.6 GPa and 9.26 MPa·m^1/2^ at 1200 °C, coinciding with the stabilization of the t-phase and grain refinement. However, further temperature elevation to 1700 °C resulted in significant grain growth (1.83 μm), leading to a decrease in hardness (7.8 GPa) consistent with the Hall–Petch effect [35]. Interestingly, at 2000 °C, the increasing content of the t- and c-phase, as confirmed by Figure 4, along with anomalous grain size refinement, contributed to a partial recovery in hardness to 11.2 GPa.

Similar trends were observed in the 5Y-PSZ and 7Y-PSZ samples, although both exhibited slightly lower fracture toughness compared to the 3Y-PSZ counterpart. For 5Y-PSZ (Figure 7b), the hardness increases with the increasing temperature as the t-phase fraction grows, reaching an inflection point at 1200 °C, where grain coarsening began to offset the strengthening effect. As the temperature rose further, the early appearance of the c-phase, due to higher Y_2_O_3_ content, led to a temporary increase in hardness, followed by a rapid decline caused by excessive grain growth and dominance of the c-phase. In 7Y-PSZ (Figure 7c), a similar trend was observed. However, the second hardness peak occurred earlier, at 1200 °C, consistent with the further reduction in the c-phase onset temperature induced by higher dopant levels. In summary, among all compositions, the 3Y-PSZ exhibited the best mechanical performance, achieving the highest hardness of 11.6 GPa and fracture toughness of 9.26 MPa·m^1/2^ at 1200 °C.

**Figure 8:**
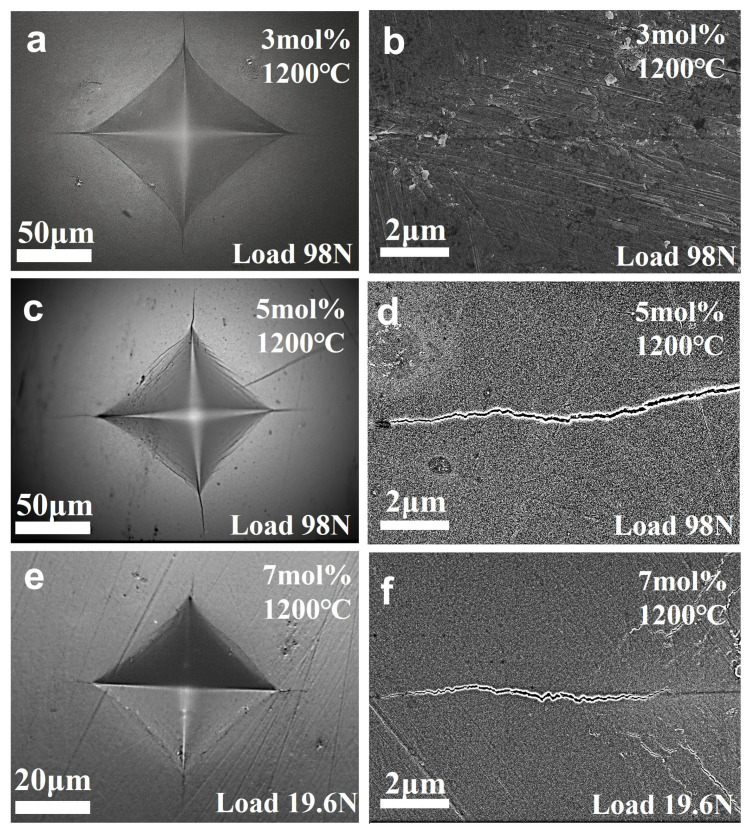
(**a**–**f**) Vickers indentations and crack propagation of (3, 5, and 7)Y-PSZ samples, sintered at 1200 °C under 5 GPa, with applied loads of 98 N, 98 N, and 19.6 N, respectively.

To further elucidate the effects of the stabilizer concentration on the fracture behavior of ZrO_2_ samples, Vickers hardness indentation and crack propagation analyses were carried out on 3Y-, 5Y-, and 7Y-PSZ samples sintered at 5 GPa and 1200 °C, as shown in Figure 8. In 3Y-PSZ, indentation under a 98 N load (Figure 8a) produced only short radial cracks. Higher magnification imaging (Figure 8b) reveals thin, blunt-ended cracks with limited extension and signs of crack branching, indicating strong resistance to crack propagation and excellent fracture toughness. The maximum toughness of 9.26 MPa·m^1/2^ achieved in this work is over 12% higher than the previously reported value of 8.2 MPa·m^1/2^ for t-phase ZrO_2_ fabricated via the TSS process [36]. This enhancement is attributed to the improved densification achieved through the HPHT method, which effectively activates the stress-induced m- to t-phase transformation toughening mechanism.

In 5Y-PSZ, the cracks are longer and more prominent (Figure 8c). A few deflected or arrested transgranular cracks are observed (Figure 8d), suggesting a partial loss of toughening mechanisms. Correspondingly, 7Y-PSZ (Figure 8e,f), exhibits a long, smooth, and uninterrupted crack path with minimal deflection or bridging, reflecting a significantly reduced crack resistance. As the Y_2_O_3_ concentration increases, the metastable t-phase becomes more stabilized and less likely to transform under stress, reducing the effectiveness of transformation toughening. In particular, the c-phase, which emerges prominently in 7Y-PSZ, is non-transformable and contributes little to fracture resistance. As a result, the contribution of transformation toughening diminishes, and cracks tend to propagate in a straighter, more brittle manner, with reduced deflection or crack-bridging effects. Quantitatively, this is reflected in the decreasing fracture toughness values: 9.26 MPa·m^1/2^ for 3Y-PSZ, 7.33 MPa·m^1/2^ for 5Y-PSZ, and 5.21 MPa·m^1/2^ for 7Y-PSZ. These observations confirm that 3Y-PSZ exhibits the highest fracture toughness among the studied compositions.

## 4. Conclusions

Nanocrystalline m-ZrO_2_ doped with 3, 5, and 7 mol% Y_2_O_3_ was successfully sintered under 5 GPa at 400 to 2000 °C, resulting in the partial or complete transformation to t- and c-phase depending on the dopant content and temperature. XRD and Raman analyses revealed that higher Y_2_O_3_ concentrations effectively reduced the transition temperatures for both t- and c-phase. SEM and hardness measurements further showed that 3Y-PSZ sintered at 1200 °C achieved the best performance, achieving a hardness of 11.6 GPa and a fracture toughness of 9.26 MPa·m^1/2^, about 12% higher than that of conventionally sintered ZrO_2_. This enhancement is attributed to pressure-induced transformation toughening. These findings demonstrate the effectiveness of HPHT processing and the critical role of Y_2_O_3_ in optimizing the hardness–toughness balance. This work contributes valuable experimental evidence for assessing the scalability and industrial applicability of the HPHT process and provides a foundation for future investigations into alternative dopant or co-doping strategies. Further studies on long-term phase stability, creep behavior, and advanced microstructural characterization are essential for fully optimizing the performance and reliability of ZrO_2_-based ceramics.

## Figures and Tables

**Table 1:** Phase composition (m: monoclinic, t: tetragonal, and c: cubic) of 3Y-PSZ ceramics under high-pressure sintering (5 GPa) at varying temperatures.

Pressure (GPa)	Temperature (°C)	Time (min)	Phase	Phase Content
5	400	10	m	m: 100%
	600		m	m: 100%
	800		m + t	m: 68% t: 32%
	1000		m + t	m: 61% t: 39%
	1200		m + t	m: 38% t: 62%
	1500		m + t	m: 38% t: 62%
	1700		m + t + c	m: 52% t + c: 47%
	2000		m + t + c	m: 30% t + c: 70%

## Data Availability

Data will be made available upon request.

## References

[1] Chevalier J., Gremillard L., Virkar A.V., Clarke D.R. (2009). The tetragonal-monoclinic transformation in zirconia: Lessons learned and future trends. J. Am. Ceram. Soc..

[2] Kelly J.R., Denry I. (2008). Stabilized zirconia as a structural ceramic: An overview. Dent. Mater..

[3] Minh N.Q. (1993). Ceramic fuel cells. J. Am. Ceram. Soc..

[4] Suk M.O., Park J.H. (2009). Corrosion behaviors of zirconia refractory by CaO–SiO_2_–MgO–CaF_2_ slag. J. Am. Ceram. Soc..

[5] Basu B., Vleugels J., Van Der Biest O. (2004). Transformation behaviour of tetragonal zirconia: Role of dopant content and distribution. Mater. Sci. Eng. A.

[6] Bocanegra-Bernal M., De La Torre S.D. (2002). Phase transitions in zirconium dioxide and related materials for high performance engineering ceramics. J. Mater. Sci..

[7] Kisi E.H., Howard C. (1998). Crystal structures of zirconia phases and their inter-relation. Key Eng. Mater..

[8] Basu B. (2005). Toughening of yttria-stabilised tetragonal zirconia ceramics. Int. Mater. Rev..

[9] Al-Khatatbeh Y., Lee K. (2014). From superhard to hard: A review of transition metal dioxides TiO_2_, ZrO_2_, and HfO_2_ hardness. J. Superhard Mater..

[10] Borik M., Bublik V., Kulebyakin A., Lomonova E., Milovich F., Myzina V., Osiko V., Tabachkova N.Y. (2014). Phase composition, structure and mechanical properties of PSZ (partially stabilized zirconia) crystals as a function of stabilizing impurity content. J. Alloys Compd..

[11] Liao S.C., Colaizzi J., Chen Y., Kear B.H., Mayo W.E. (2000). Refinement of Nanoscale Grain Structure in Bulk Titania via a Transformation-Assisted Consolidation (TAC) Method. J. Am. Ceram. Soc..

[12] Piconi C., Maccauro G. (1999). Zirconia as a ceramic biomaterial. Biomaterials.

[13] Tel H., Altaş Y., Eral M., Sert Ş., Çetinkaya B., İnan S. (2010). Preparation of ZrO_2_ and ZrO_2_–TiO_2_ microspheres by the sol–gel method and an experimental design approach to their strontium adsorption behaviours. Chem. Eng. J..

[14] Wang J., Chu D., Ma H., Fang S., Chen Q., Liu B., Ji G., Zhang Z., Jia X. (2021). Effect of sintering temperature on phase transformation behavior and hardness of high-pressure high-temperature sintered 10 mol% Mg-PSZ. Ceram. Int..

[15] Xia Y., Mou J., Deng G., Wan S., Tieu K., Zhu H., Xue Q. (2020). Sintered ZrO_2_–TiO_2_ ceramic composite and its mechanical appraisal. Ceram. Int..

[16] Yang Y., Hu C., Liu Q., Li J. (2024). Research progress and prospects of colored zirconia ceramics: A review. J. Adv. Ceram..

[17] Denry I., Kelly J.R. (2008). State of the art of zirconia for dental applications. Dent. Mater..

[18] Alhotan A., Yilmaz B., Weber A., Babaier R., Bourauel C., Fouda A.M. (2024). Effect of artificial aging on fracture toughness and hardness of 3D-printed and milled 3Y-TZP zirconia. J. Prosthodont..

[19] Tovar-Vargas D., Roitero E., Anglada M., Jiménez-Piqué E., Reveron H. (2021). Mechanical properties of ceria-calcia stabilized zirconia ceramics with alumina additions. J. Eur. Ceram. Soc..

[20] Kambale K., Mahajan A., Butee S. (2019). Effect of grain size on the properties of ceramics. Met. Powder Rep..

[21] Lu K., Lei Z., Deng S., Li J., Feng T., Luo Z., Ma X. (2023). Synergistic effects of grain sizes on the corrosion behavior and mechanical properties in a metastable high-entropy alloy. Corros. Sci..

[22] Raj R. (1987). Analysis of the sintering pressure. J. Am. Ceram. Soc..

[23] Wilkinson D.S., Ashby M. (1975). Pressure sintering by power law creep. Acta Metall..

[24] Gao L., Li W., Wang H., Zhou J., Chao Z., Zai Q. (2001). Fabrication of nano Y–TZP materials by superhigh pressure compaction. J. Eur. Ceram. Soc..

[25] Munoz-Saldana J., Balmori-Ramirez H., Jaramillo-Vigueras D., Iga T., Schneider G. (2003). Mechanical properties and low-temperature aging of tetragonal zirconia polycrystals processed by hot isostatic pressing. J. Mater. Res..

[26] Vahldiek F., Robinson L., Lynch C. (1963). Tetragonal zirconium oxide prepared under high pressure. Science.

[27] Kulcinski G. (1968). High-Pressure Induced Phase Transition in ZrO_2_. J. Am. Ceram. Soc..

[28] Li X., Wang Y., Wang F., Liang A. (2021). Ta_2_O_5_ in-situ composite Ta-based nanocrystalline coating with wonderful wear resistance and related wear mechanisms. Mater. Lett..

[29] Toraya H., Yoshimura M., Somiya S. (1984). Calibration curve for quantitative analysis of the monoclinic-tetragonal ZrO_2_ system by X-ray diffraction. J. Am. Ceram. Soc..

[30] Brito-Chaparro J., Reyes-Rojas A., Bocanegra-Bernal M., Aguilar-Elguezabal A., Echeberria J. (2007). Elucidating of the microstructure of ZrO_2_ ceramics with additions of 1200° C heat treated ultrafine MgO powders: Aging at 1420 °C. Mater. Chem. Phys..

[31] Feinberg A., Perry C. (1981). Structural disorder and phase transitions in ZrO_2_-Y_2_O_3_ system. J. Phys. Chem. Solids.

[32] Michel D., Collongues R. (1976). Study by Raman spectroscopy of order-disorder phenomena occurring in some binary oxides with fluorite-related structures. J. Raman Spectrosc..

[33] Calderon-Moreno J.M., Yoshimura M. (2002). Characterization by Raman spectroscopy of solid solutions in the yttria-rich side of the zirconia–yttria system. Solid State Ion..

[34] Sekulić A., Furić K., Stubičar M. (1997). Raman study of phase transitions in pure and alloyed zirconia induced by ball-milling and a laser beam. J. Mol. Struct..

[35] Dunstan D., Bushby A. (2014). Grain size dependence of the strength of metals: The Hall–Petch effect does not scale as the inverse square root of grain size. Int. J. Plast..

[36] Chee H.A., Singh R., Lee K.S. (2021). Effects of pressureless two-step sintering on the densification and properties of tetragonal zirconia. J. Ceram. Process. Res..

